# gmx_ffconv: A Fast, User-Friendly Semi-Automated All-Atom Force Field Converter for GROMACS

**DOI:** 10.1021/acs.jcim.5c02200

**Published:** 2025-09-20

**Authors:** Jasmine E. Aaltonen

**Affiliations:** Department of Chemistry, 4396 Lancaster University, Lancaster LA1 4YB, U.K.

## Abstract

This application note presents gmx_ffconv, a command-line tool developed to facilitate the conversion of systems between all-atom force fields within GROMACS. As different force fields use their own naming conventions and atom ordering, force field conversion within GROMACS is usually a time-consuming, error-prone process. gmx_ffconv resolves atom ordering and naming mismatches between different force fields by reordering the coordinate file via molecular graph matching. This enables the use of identical starting coordinates across force fields, facilitating comparative simulations without requiring manual reordering or scripting. The tool has been validated on a broad range of systems, from small, nonstandard ligands to large, solvated heterogeneous systems with more than two million atoms. gmx_ffconv is available on GitHub: github.com/Jassu1998/gmx_ffconv.

## Graphical abstract

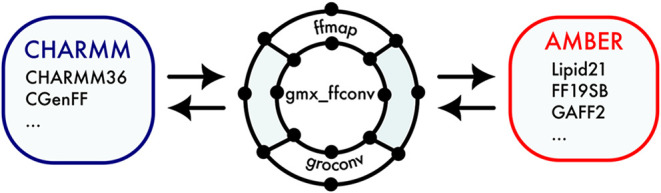

## Introduction

Molecular dynamics (MD) simulations are gaining popularity with the increase in computational power and are widely used for modeling biological systems, chemical phenomena and materials.^1–4^ GROMACS is one of the most widely used MD codes, known for excellent performance of large systems such as biological ones.^5^ In MD simulations, systems are described with force fields that define their potential energy using a collection of equations and parameters.^6^ The two most notable families of force fields for biomolecular simulations are CHARMM (Chemistry at HARvard Macromolecular Mechanics) and AMBER (Assisted Model Building and Energy Refinement).^7,8^ Even widely used robust force fields, such as CHARMM and AMBER, exhibit differences in predicted properties, such as the solubility of functional groups and the relative stability of protein conformations.^9–11^ The importance of force field choice and validation is well-established.^12,13^ Ideally, one would validate against experimental data and if that is not available, against other force fields.^14^ However, this is the stage when users often encounter difficulties due to a lack of tools which support the conversion of GROMACS input files from one force field to another.

Unlike existing tools, gmx_ffconv enables direct force field conversion by reordering GROMACS coordinate files, without requiring CHARMM inputs or format translation. Currently, the options for users to convert force fields include CHARMM-GUI’s Force Field Converter and the built-in tool in GROMACS, pdb2gmx.^15–17^ The downside of using Force Field Converter is the prerequisite of CHARMM input files (requires a CHARMM topology file (.psf) and CHARMM coordinate file (.crd)), which therefore requires users to first convert their inputs to CHARMM. Furthermore, Force Field Converter is limited to converting CHARMM-compatible molecules to the two supported force fields, AMBER and CHARMM.^15^ Additionally, if the new force field contains molecules that are not in the database, Force Field Converter will terminate with an error. The alternative is to use pdb2gmx, which usually requires users to alter the residue topology (.rtp) files stored in GROMACS and ensure the syntax used in the coordinate file matches the residue topology files. For example, even a lipid as standard as DPPC cannot be directly converted from an AMBER Lipid21-formatted coordinate file to a CHARMM36 coordinate file via pdb2gmx. This is due to mismatches in residue and atom names, as well as atom ordering.

In order to carry out a simulation in GROMACS, in addition to run parameters, the user must provide an input coordinate file and a corresponding topology file. Unlike other MD codes, GROMACS requires the order of atoms in the coordinate file to match exactly the order used in the topology. The most common coordinate file format used in GROMACS is the Gromos87 file format (.gro, see Section S1.1 for an example of a coordinate file), usually simply referred to as a GRO file. The GRO file is a text file that follows a fixed format, with a title string, followed by the number of atoms, a line for each atom and the final line containing the simulation box vectors. Each atom line needs to contain a residue number, residue name, atom name, atom number, coordinates and (optional) velocities. In GROMACS, topologies are typically built using a main topology file (.top), which describes the system and references additional topology files (.itp), called included topology files (ITP files).

ITP files are text files that contain general force field parameters or molecule descriptions. For example, a typical nonsolvent small molecule topology file will contain multiple blocks, describing the type of molecule, the atoms, the bonds, the angles etc. (see Section S1.1 for an example of an included topology file).

Currently, one of the main ways users prepare all-atom systems is via the use of CHARMM-GUI. CHARMM-GUI is a web server that enables the preparation of a variety of systems, such as bilayers with embedded proteins and vesicles among many others.^18^ In cases where the required molecules are absent from the CHARMM-GUI database, tools like PACKMOL, which support user-defined packing, or in-house preparation methods are commonly used.^19^ In addition to the possibility of obtaining the ITP files via CHARMM-GUI, there exist numerous tools to create ITP files for unparametrized molecules, such as CGenFF for CHARMM and AcPype for AMBER.^20,21^ There is a notable number of software packages that enable users to convert between simulation software, such as Parmed for AMBER to GROMACS and TopoGromacs for CHARMM to GROMACS.^22,23^ However, they do not support force field conversion; the force field must be maintained across simulation codes. While manually converting very small molecules may be feasible, the process quickly becomes impractical as molecular size increases (see Section S1.2). Furthermore, simulated systems often consist of hundreds of thousands of atoms, which would require the user to determine the correct atom ordering for each molecule and produce a custom script to realign the whole system with the new force field.

gmx_ffconv is designed for force field developers and practitioners who want to compare different parametrizations or force fields. It also enables users to easily convert complex, pre-equilibrated systems from literature or other sources into their preferred force field. Currently, gmx_ffconv supports GROMACS coordinate files (.gro) and included topology (.itp) files. The conversion of systems between force fields in gmx_ffconv (Figure 1) is done via the use of two utilities: ffmap, which finds a mapping across the two force fields and groconv, which performs the conversion of the coordinate file to match the topology of the new force field. It is important to note that gmx_ffconv does not create or modify force field parameters, nor does it perform any form of physical validation of the resulting systems.

**Figure 1:**
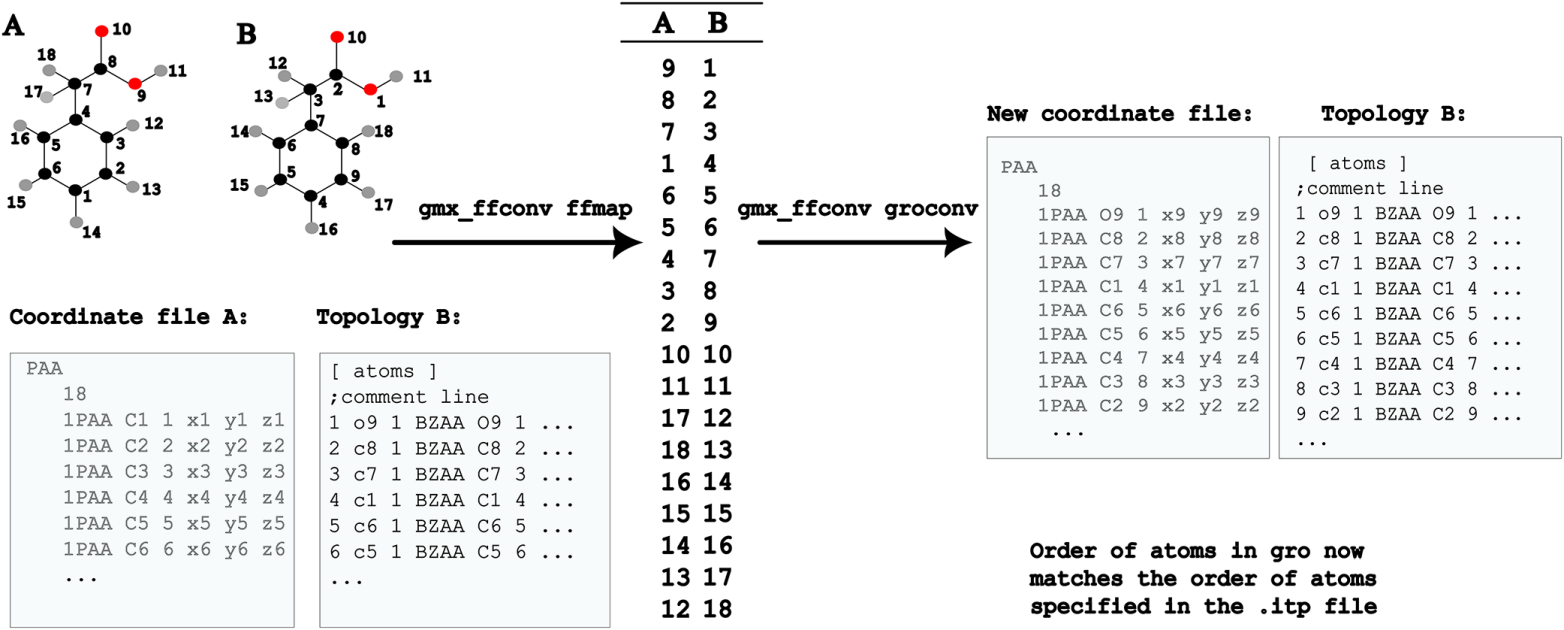
Schematic illustration of the gmx_ffconv workflow for converting a system between two force fields. First, a mapping is found between the two force fields for each type of molecule via ffmap. Then, the original coordinate file is reordered to match the ordering used in the new force field via groconv.

## Implementation

### ffmap

As there is no standard practice when it comes to naming atoms or even residues across force fields, the systems cannot be aligned using atom or residue names. Furthermore, residues are sometimes split into multiple residues depending on the force field, making a residue-based approach infeasible. Instead of a templating-based approach, gmx_ffconv uses molecular graphs. A molecular graph is a connected graph where atoms are represented as labeled nodes and bonds as edges. The ffmap utility reads from the included topology files the atoms and bonds blocks. For each atom, ffmap identifies the chemical element from the atomic mass in the included topology file. The chemical element is assigned to the closest match; however, ffmap terminates if no match is found within 0.3 atomic mass units. As classical force fields treat all bonds as harmonic springs, each bond corresponds to a single edge in the graph. For molecules that do not contain a bonds block, such as rigid water models that are handled via the SETTLE algorithm, the [ settles ] block is used instead.^24^

To run ffmap, the user must simply provide a name for the molecule being converted (-name), and the paths to the two ITP files, the one for the current force field (-itp1) and the new force field (-itp2). The name provided by the user does not need to match the residue name used in either one of the included topology files. Furthermore, as gmx_ffconv does not use templating, no atom or residue names need to be modified by the user. A labeled graph is constructed for each ITP file. First, labeled nodes with index and element type are created. Next, the nodes are connected based on the bonds described in the respective bonds or settles blocks. A graph isomorphism, essentially a mapping, is run between the two graphs using the variant of VF2 implemented in NetworkX.^25–27^ Very briefly, VF2 is a step-by-step backtracking algorithm that builds a mapping between two graphs by adding nodes only when they satisfy feasibility rules, which include both syntactic conditions (like matching connectivity) and semantic conditions (like matching node types). Importantly, if an isomorphism exists (in other words, if the molecules are the same), the algorithm, by design, is guaranteed to find a mapping between the graphs.^27^ The default behavior in gmx_ffconv is to return the first isomorphism found. If the user provides two topologies that do not correspond to the same molecule, gmx_ffconv will return an error message informing the user that the graphs are not isomorphic.

Graph isomorphisms and their time complexity are important topics in computer science; however, they are beyond the scope of this application note.^28,29^ However, it should be noted that the scaling of ffmap does not simply depend on the number of atoms, but also on how much symmetry a molecule has, with higher symmetry molecules taking longer. For reasonably sized molecules (less than 200 atoms), the mapping is typically fast, with time scales ranging from less than a second to minutes on modern hardware (Table 2). In the case of large molecules, such as proteins, typically mappings remain fast as proteins tend to consist of multiple chains that are mapped individually. Even for very large proteins, the longest mappings took under 10 min (Table 3). Inconsistencies in protonation states will prevent ffmap from finding a mapping. For example, histidine residues adopt different protonation states depending on pH. If ffmap requires more than 10 min to complete, this generally indicates a protonation mismatch in the input files that should be addressed by the user.

While the current approach successfully identifies a mapping between two graphs, it introduces a secondary consideration arising from the indistinguishability of hydrogen atoms bonded to the same carbon. In the case of chemical equivalence, this approach will not cause issues (see Section S2 for further discussion). In all of the tested validation systems (Table 2), hydrogens were treated as chemically equivalent, and no mapping failures were encountered. Diastereotopic hydrogens, however, are not chemically equivalent. They are commonly found on carbons adjacent to stereocenters, for example, in protein side chains. Nevertheless, standard force fields do not differentiate between them during parametrization. Consequently, in typical molecular dynamics simulations, swapping the order of diastereotopic hydrogens does not affect bonded or nonbonded interactions. While the permutation of hydrogens does not affect the validity of MD simulations, it can impact analyses that rely on consistent atom names.

To address this, the latest version of gmx_ffconv (v1.0.3) supports consistent atom naming across force fields. Users who wish to ensure consistent atom naming across force fields must provide a CSV file containing atom name equivalencies to ffmap via the --consistent_naming flag. The CSV must include a header, with atom names from the current force field in the first column and the corresponding names from the new force field in the second. Only atom names included in the CSV are explicitly considered during mapping; all others are mapped based on chemical element and connectivity. If an atom name in the original force field corresponds to multiple atom names in the target force field, the names must be temporarily made unique for the mapping (see Supporting Information, Example 3).

Since gmx_ffconv transforms the coordinate file to match the target force field, this ensures that restraints included in the new force field topology files are correctly applied. Features such as disulfide bridges are supported only if present in both topologies. Restraint atom indices must match the ordering used in the updated coordinate file to be properly evaluated by GROMACS. If this condition is not met, restraint files must either be regenerated or converted. Conversion is generally easier than regeneration and can be accomplished using conversion scripts that renumber atom indices to align with the new force field. A script for converting position restraints is provided in the Supporting Information. A script for NMR restraints generated by nmr2gmx is currently available on GitHub, and additional scripts will be provided upon request.^30^

### groconv

groconv reads the original gro file (-coordfile) and reorganizes each molecule individually, as specified by the user with -name and -nmol (the molecule names and their respective counts, separated by spaces). The names and counts must match the order used in the original coordinate file. For each molecule type, the program uses the corresponding mapping file to reorder the atoms to match the order used in the new force field. By default, groconv outputs a reordered gro file (-output) in which residues are automatically renamed and renumbered to match the new force field. The recommended practice for using gmx_ffconv is to build a database of mappings and storing them in a dedicated directory, which can be provided to groconv via the -mapping_dir option. To facilitate the automated renaming of residues and atoms, the included topologies for the new force field should also be stored in the mappings directory, as groconv will search there automatically. If the necessary files are not found, users will be prompted to provide their paths manually.

### Validation

To validate the mapping and conversion, a back-conversion approach has been implemented in gmx_ffconv. It should be noted that gmx_ffconv is designed to be robust, and users are not expected to perform validation routinely. However, validation is recommended for suspected edge cases or if the resulting systems exhibit instability. The back-converted structure can be obtained by adding the --validate flag to the ffmap and groconv commands. Since the back-converted structure may differ due to hydrogen permutation and possible reordering of chemical groups, rather than comparing the coordinate files directly, a single-point energy should be calculated instead. If the conversion is successful, all energy values (bonded and unbonded) reported in the log files are identical between the original structure and the back-converted one. An alternative approach is to compare the structures via gmx rms, which returns a deviation of zero if the structures are the same. The validation systems were chosen to illustrate diverse use cases, including phenylacetic acid as a cyclic, nonstandard ligand, a large viral membrane containing various lipids, standard proteins such as human serum albumin (HSA, Protein Data Bank ID: 1AO6), and a less common use case involving a glycosylated protein (Protein Data Bank ID: 6VSB). All systems (Table 1) were prepared using CHARMM-GUI.^17,18,31,32^ For CHARMM-based systems, the CHARMM36m force field was used for proteins and lipids, and CGenFF for small molecules.^7,20^ For AMBER-based systems, the ff19SB force field was used for proteins, the Lipid21 force field for lipids and GAFF2 for small molecules.^8,33,34^ Additional details regarding system setup, abbreviations and the validation approach are provided in the Supporting Information (Section S2).

**Table 1:** Description of Systems Used For Validation of gmx_ffconv

system	molecule name	number of molecules	number of atoms
phenylacetic acid	BZAA	1	18
viral membrane	CHL	705	52,170
	DPPC	94	12,220
	PSM	470	59,690
	DPPE	282	34,122
	POPE	423	52,875
	POPC	141	18,894
	POPS	141	17,907
	DPPS	94	11,562
	K^+^	2128	2128
	Cl^–^	1857	1857
	TIP3P	668,899	2,006,697
	**Total**	**675,234**	**2,270,122**
human serum albumin (HSA)	PROA	1	9123
	PROB	1	9123
	**Total**	**2**	**18,246**
fully glycosylated SARS-coV-2 spike protein	PROA	1	24,331
	PROB	1	24,330
	PROC	1	24,329
	**Total**	**3**	**72,990**

### Performance

All timing measurements in Tables 2 and 3 were performed on a M1 MacBook Air using gmx_ffconv v1.0.2, Python v3.9, NetworkX v2.8.5, and NumPy v2.0.2.^26,35,36^ The timings in Table 2 were measured using the shell built-in time command and repeated 5 times to obtain an average and sample standard deviations. The 95% confidence intervals, assuming a Student’s t-distribution, were calculated using Excel.^37^ It should be noted that despite the timings of some mappings not passing the Shapiro-Wilk test for normality, this possible deviation from normality does not affect the conclusions.^38^

**Table 2:** Wall-Clock Timings (In Seconds) for gmx_ffconv’s ffmap Utility, Showing Mean Values with 95% Confidence Intervals for a Small Molecule, Lipids and Proteins

molecule	CHARMM → AMBER	AMBER → CHARMM
BZAA	0.10 [0.09, 0.10]	0.10 [0.09, 0.10]
CHL	0.10 [0.10, 0.10]	0.10 [0.10, 0.10]
DPPC	65.48 [64.71, 66.25]	0.11 [0.10, 0.11]
DOPE	60.02 [59.81, 60.23]	0.33 [0.32, 0.33]
HSA chain A	48.79 [47.86, 49.71]	49.02 [48.05, 49.99]
HSA chain B	48.54 [47.58, 49.49]	48.55 [48.15, 48.96]

**Table 3:** Wall-Clock Timing Breakdown of gmx_ffconv’s ffmap and groconv Utilities for Validation Systems^a^

system	molecule name	ffmap	groconv
phenylacetic acid	BZAA	0.09	0.09
viral membrane	CHL	0.27	
	DPPC	66.32	
	PSM	7.14	
	DPPE	67.33	
	POPE	0.36	
	POPC	0.26	
	POPS	0.25	
	DPPS	65.61	
	K^+^	0.11	
	Cl^–^	0.09	
	TIP3P	0.19	
	**Total**	207.92^b^	4.47
		71.31^c^	
fully glycosylated SARS-coV-2 spike protein	PROA	413.79	
	PROB	409.42	
	PROC	400.79	
	**Total**	1224.00^b^	0.40
		492.76^c^	

a All times are reported in seconds to two decimal places.

b Run sequentially.

c Run simultaneously.

The performance is excellent, with the slower systems taking up to a minute (Table 2). As VF2 is a deterministic algorithm, the time to resolution for two labeled graphs is constant, as evidenced by the narrow 95% confidence intervals in Table 2. This implies that restarting ffmap will not yield a faster solution. It is, however, possible that the matching algorithm finds a solution faster the other way around, for example, for DPPC from AMBER to CHARMM takes almost 650 times longer than from CHARMM to AMBER. In principle, users can provide the ITP files in reverse order, however, this would also require manual alteration of the mappings file (swapping the columns and resorting the second column in increasing order). While this approach should not be necessary for most users, it may be worth considering if ffmap is taking an unusually long time and the user is certain there are no differences in protonation. The difference in performance stems from how the nodes are ordered, affecting the search process and thus speed. This sensitivity to node ordering can also be observed in Table 2, where timings do not follow or scale as a function of molecular size. It should be noted here that the user may launch multiple instances of ffmap to find mappings for all the types of molecules simultaneously, instead of sequentially. Fortunately, the bottleneck in gmx_ffconv is the ffmap stage (Table 3). This enables the possibility for users to build a database with all their mappings instead of having to run ffmap repeatedly. If a molecule is missing from the database, the user can simply run ffmap for that specific molecule, add it to the database and proceed as standard. By building a database of mappings, by simply storing the mappings in a directory, it is possible to perform the conversion in a matter of seconds for most systems (Table 3).

## Conclusions

To conclude, gmx_ffconv provides a streamlined and automated approach for converting molecular systems between different force fields within the GROMACS ecosystem. By removing the need for custom scripting and manual file manipulation, gmx_ffconv lowers the technical barrier of performing force field conversions, facilitating broader adoption of comparative validation studies. Only two conditions must be fulfilled: the user must have included topologies for each type of molecule in the two force fields, and the protonation must be consistent across force fields, tautomers are not supported. When these criteria are met, gmx_ffconv can efficiently convert the original coordinate file to match the new force field.

However, gmx_ffconv does have limitations, with the biggest limitation being the lack of support for water model conversion, specifically from 3-point to 4-point models or vice versa. Additionally, as protonation states must remain consistent across force fields, transformations involving changes in protonation are not supported. These transformations fall outside the current scope of gmx_ffconv, as they would require the generation of new coordinates for missing virtual sites (such as dummy particles present in 4-point water models) or hydrogens introduced by changes in protonation states. Nevertheless, systems can be readily resolvated using utilities such as GROMACS’s built-in solvate tool, allowing users to regenerate the solvent environment with the new water model. Overall, gmx_ffconv is predicted to be robust across a variety of use cases and will promote an increase in system validation across force fields.

## Supplementary Material





## Data Availability

gmx_ffconv is freely available on GitHub (github.com/Jassu1998/gmx_ffconv) and archived via Zenodo (DOI: 10.5281/zenodo.16739221). All software and data used in this manuscript, including topology and coordinate files, raw data for Tables 2 and 3, statistical testing and conversion script for position restraints are provided in the Supporting Information ZIP file (gmx_ffconv.zip).

## Notes

Example coordinate file and included topology file. Also contains molecular graphs for DPPC (CHARMM36 and AMBER Lipid21) (Section S1); description of how simulation systems were obtained from CHARMM-GUI and setup, discussion behind validation approach and abbreviations for all molecules (Section S2);installation instructions and example cases of usage (Section S3) (PDF)
Contains a README and the version of gmx_ffconv used in this manuscript for timings and the latest version. Contains all the topology files and coordinate files used for this manuscript, validation data and a conversion script for position restraints; an Excel file with the raw data used in the Tables 2 and 3 and statistical testing; “Tutorial” folder contains the files described in section 3 of SI_gmx_ffconv (ZIP)
