# Supplementary material for: gmx_ffconv: A Fast, User-Friendly Semi-Automated All-Atom Force Field Converter for GROMACS
Source: J Chem Inf Model. 2025 Sep 20;65(19):9850–5. doi: 10.1021/acs.jcim.5c02200 (PMC12529758; doi:10.1021/acs.jcim.5c02200)
Supplement: Supplementary file 2 [file ci5c02200_si_002.zip › gmx_ffconv_JCIM/Tutorial/SI_gmx_ffconv.pdf]

Supplementary Information

gmx_ffconv: A Fast, User-Friendly Semi-Automated All-Atom Force Field Converter for GROMACS

Jasmine E. Aaltonen[a*]

[a]*Department of Chemistry, Lancaster University, Lancaster, LA1 4YB, United Kingdom*

E-mail: j.aaltonen@lancaster.ac.uk

Contents

1.1 Examples of coordinate and included topology files 8

1.2 Molecular connectivity graphs of DPPC 10

2. Validation approach for conversion: Forwards and backwards conversion 10

Rationale behind using a forwards and backwards conversion approach 11

How sensitive are the energies printed in the log file? 12

Simulation set-up 12

Abbreviations used in Table 2 13

Test case: (Lack of) effect of incorrect hydrogen ordering 13

3. User manual for gmx_ffconv 15

Installation instructions 15

Example usage of gmx_ffconv 16

1.1 Examples of coordinate and included topology files

Phenylacetic acid (BZAA) was obtained from CHARMM-GUI using Ligand Reader and Force Field Converter. The gro file's last line has been modified to contain only the first 3 simulation box vectors.

BZAA_CHARMM.gro, coordinate file

```
Generated by CHARMM-GUI FF-Converter
18
    1BZAA C1 1 0.201 0.119 0.023
    1BZAA H1 2 0.274 0.144 0.099
    1BZAA C2 3 0.192 0.195 -0.094
    1BZAA H2 4 0.258 0.279 -0.109
    1BZAA C3 5 0.096 0.163 -0.192
    1BZAA H3 6 0.087 0.223 -0.281
    1BZAA C4 7 0.010 0.054 -0.172
    1BZAA H4 8 -0.066 0.031 -0.246
    1BZAA C5 9 0.020 -0.023 -0.056
    1BZAA C6 10 0.115 0.010 0.042
    1BZAA H6 11 0.121 -0.048 0.133
    1BZAA C7 12 -0.078 -0.135 -0.030
    1BZAA H71 13 -0.029 -0.213 0.031
    1BZAA H72 14 -0.107 -0.180 -0.128
    1BZAA C8 15 -0.207 -0.091 0.042
    1BZAA O8 16 -0.297 -0.167 0.071
    1BZAA O9 17 -0.219 0.043 0.076
    1BZAA H9 18 -0.303 0.055 0.119
   0.00000 0.00000 0.00000
```

BZAA_CHARMM.itp, included topology file

```
;;
;; Generated by CHARMM-GUI FF-Converter
;;
;; Correspondance:
;; jul316@lehigh.edu or wonpil@lehigh.edu
;;
;; GROMACS topology file for BZAA
;;


[ moleculetype ]
; name nrexcl
BZAA 3

[ atoms ]
; nr type resnr residu atom cgnr charge mass
    1 CG2R61 1 BZAA C1 1 -0.115000 12.0110 ; qtot -0.115
    2 HGR61 1 BZAA H1 2 0.115000 1.0080 ; qtot 0.000
    3 CG2R61 1 BZAA C2 3 -0.115000 12.0110 ; qtot -0.115
    4 HGR61 1 BZAA H2 4 0.115000 1.0080 ; qtot 0.000
    5 CG2R61 1 BZAA C3 5 -0.115000 12.0110 ; qtot -0.115
    6 HGR61 1 BZAA H3 6 0.115000 1.0080 ; qtot 0.000
    7 CG2R61 1 BZAA C4 7 -0.105000 12.0110 ; qtot -0.105
    8 HGR61 1 BZAA H4 8 0.115000 1.0080 ; qtot 0.010
    9 CG2R61 1 BZAA C5 9 -0.000000 12.0110 ; qtot 0.010
   10 CG2R61 1 BZAA C6 10 -0.105000 12.0110 ; qtot -0.095
   11 HGR61 1 BZAA H6 11 0.115000 1.0080 ; qtot 0.020
   12 CG321 1 BZAA C7 12 -0.200000 12.0110 ; qtot -0.180
   13 HGA2 1 BZAA H71 13 0.090000 1.0080 ; qtot -0.090
   14 HGA2 1 BZAA H72 14 0.090000 1.0080 ; qtot 0.000
   15 CG2O2 1 BZAA C8 15 0.720000 12.0110 ; qtot 0.720
   16 OG2D1 1 BZAA O8 16 -0.550000 15.9994 ; qtot 0.170
```

```
    17 OG311 1 BZAA O9 17 -0.600000 15.9994 ; qtot -0.430
    18 HGP1 1 BZAA H9 18 0.430000 1.0080 ; qtot -0.000

[ bonds ]
; ai aj funct b0 Kb
    1 2 1
    3 1 1
   10 1 1
    3 4 1
    5 3 1
    6 5 1
    7 5 1
    8 7 1
    7 9 1
    9 10 1
   12 9 1
   10 11 1
   12 13 1
   14 12 1
   15 12 1
   16 15 1
   15 17 1
   17 18 1

[ pairs ]
; ai aj funct c6 c12 or
; ai aj funct fudgeQQ q1 q2 c6 c12
    1 6 1
    1 7 1
    1 12 1
    2 4 1
    2 5 1
    2 9 1
```

```
   2 11 1
   3 8 1
   3 9 1
   3 11 1
   4 6 1
   4 7 1
   4 10 1
   5 10 1
   5 12 1
   6 8 1
   6 9 1
   7 11 1
   7 13 1
   7 14 1
   7 15 1
   8 10 1
   8 12 1
   9 16 1
   9 17 1
  10 13 1
  10 14 1
  10 15 1
  11 12 1
  12 18 1
  13 16 1
  13 17 1
  14 16 1
  14 17 1
  16 18 1

[ angles ]
; ai aj ak funct th0 cth S0 Kub
   2 1 3 5
```

```
   2 1 10 5
   3 1 10 5
   1 3 4 5
   1 3 5 5
   4 3 5 5
   3 5 6 5
   3 5 7 5
   6 5 7 5
   5 7 8 5
   5 7 9 5
   8 7 9 5
   7 9 10 5
   7 9 12 5
  10 9 12 5
   1 10 9 5
   1 10 11 5
   9 10 11 5
   9 12 13 5
   9 12 14 5
   9 12 15 5
  13 12 14 5
  13 12 15 5
  14 12 15 5
  12 15 16 5
  12 15 17 5
  16 15 17 5
  15 17 18 5

[ dihedrals ]
; ai aj ak al funct phi0 cp mult
   2 1 3 4 9
   2 1 3 5 9
   2 1 10 9 9
```

```
 2 1 10 11 9
 3 1 10 9 9
 3 1 10 11 9
 4 3 1 10 9
 5 3 1 10 9
 1 3 5 6 9
 1 3 5 7 9
 4 3 5 6 9
 4 3 5 7 9
 3 5 7 8 9
 3 5 7 9 9
 6 5 7 8 9
 6 5 7 9 9
 5 7 9 10 9
 5 7 9 12 9
 8 7 9 10 9
 8 7 9 12 9
 7 9 10 11 9
 7 9 12 13 9
 7 9 12 14 9
 7 9 12 15 9
10 9 12 13 9
10 9 12 14 9
10 9 12 15 9
 1 10 9 7 9
 1 10 9 12 9
11 10 9 12 9
 9 12 15 16 9
 9 12 15 17 9
13 12 15 16 9
13 12 15 17 9
14 12 15 16 9
14 12 15 17 9
```

```
  12 15 17 18 9

  16 15 17 18 9


[ dihedrals ]

; ai aj ak al funct q0 cq

  15 12 16 17 2


#ifdef POSRES

[ position_restraints ]

   1 1 POSRES_FC_BB POSRES_FC_BB POSRES_FC_BB

   3 1 POSRES_FC_BB POSRES_FC_BB POSRES_FC_BB

   5 1 POSRES_FC_BB POSRES_FC_BB POSRES_FC_BB

   7 1 POSRES_FC_BB POSRES_FC_BB POSRES_FC_BB

   9 1 POSRES_FC_BB POSRES_FC_BB POSRES_FC_BB

  10 1 POSRES_FC_BB POSRES_FC_BB POSRES_FC_BB

  12 1 POSRES_FC_BB POSRES_FC_BB POSRES_FC_BB

  15 1 POSRES_FC_BB POSRES_FC_BB POSRES_FC_BB

  16 1 POSRES_FC_BB POSRES_FC_BB POSRES_FC_BB

  17 1 POSRES_FC_BB POSRES_FC_BB POSRES_FC_BB

#endif
```

1.2 Molecular connectivity graphs of DPPC

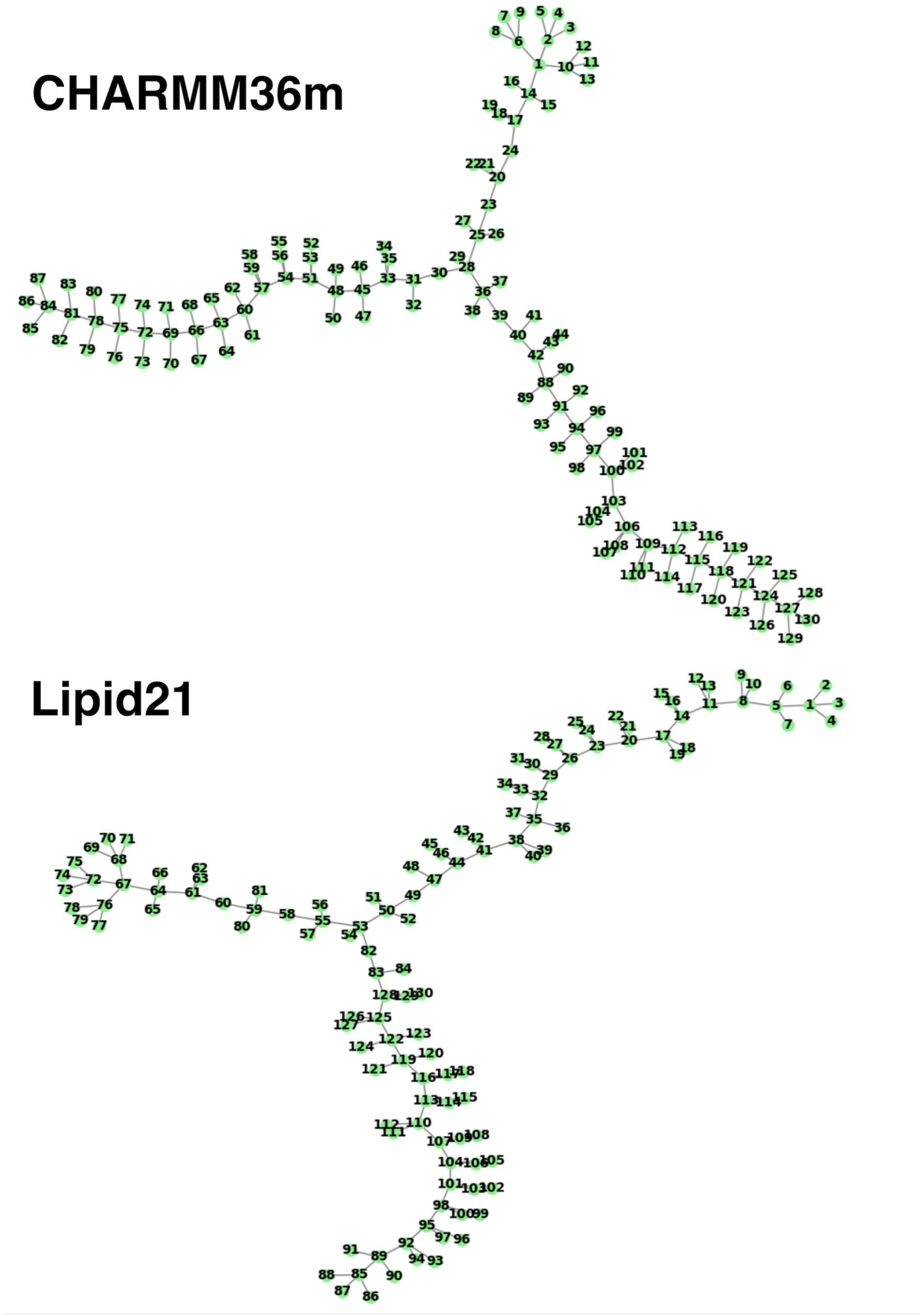

Figure S1: Connectivity graphs of DPPC in two force fields: CHARMM36m and Lipid21 (AMBER).

2. Validation approach for conversion: Forwards and backwards conversion

Rationale behind using a forwards and backwards conversion approach

The big challenge in coming up with a validation approach for gmx_ffconv is the fact that in the typical use case, the user will have the system coordinates for a force field and only topologies for both force fields. During the development of gmx_ffconv, systems could be compared and validated against those obtained from the Force Field Converter implemented in CHARMM-GUI. However, in most cases users will not have the CHARMM formatted input files and/or have molecules that are not in the database. In theory, when a system is converted from a force field A to a force field B, and then that new coordinate file is converted back using mappings obtained from B to A, this should result in the initial structure (up to permutation). In the case of an incorrect mapping being found, the same incorrect mapping would need to be found in both directions for this to error-cancel. The user can also visually inspect the mapping files and compare them with labelled molecules, for example by extracting a molecule and visualising it in pymol with atom labels.

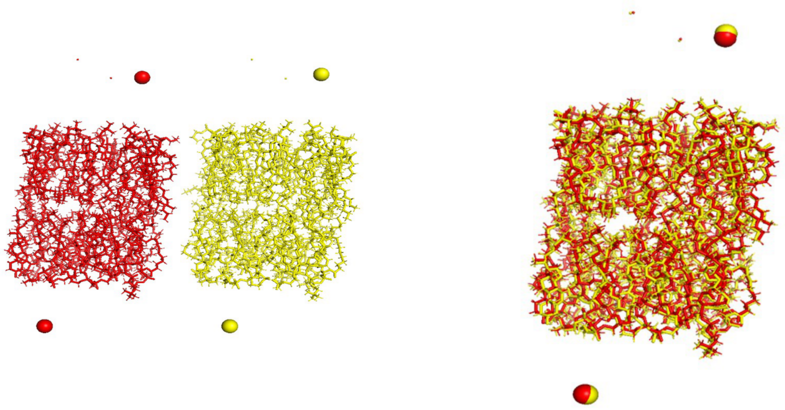

Figure S2: Small membrane containing DPPC, CHL and DOPE obtained from CHARMM-GUI.

As CHARMM-GUI is one of the most widely used approaches for membrane generation, it makes for the perfect validation target. However, even creating two identical systems with CHARMM-GUI can be difficult. For example, when creating a membrane with the bilayer tool, the user must specify a force field and the next step gives the files to download. If the user goes back, selects another force field and return to the next step the new systems should in principle match. However, there is minor (< 0.01 nm) unsystematic offset between all the new coordinates compared to the previous force field (Fig. S2). If instead the user were to use the last .psf and .crd included in the output given by CHARMM-GUI and use Force Field Converter in order to get a matching system, it is not sufficient to merely take the box size from the gro file, instead the box size must be extracted from one of the CHARMM-GUI files, including all decimal places.

Example 2 shows an example where energies were validated against a structure converted by CHARMM-GUI.

How sensitive are the energies printed in the log file?

It is a reasonable question to ask if comparing energies from before and after the conversion would flag up an error in the conversion. To test this, the system used in Example 1 with 16 131 atoms was used. The first atom's x-coordinate was displaced by 0.001 (2.544 to 2.543).

Table S1: Comparison of energy components between original and displaced configurations.

Quantity	Original Configuration	Displaced Configuration
Bond Energy (kJ/mol)	$7.14729 \cdot 10^3$	$7.14744 \cdot 10^3$
Urey-Bradley	$5.62603 \cdot 10^4$	$5.62597 \cdot 10^4$
Proper Dihedral	$1.41737 \cdot 10^4$	$1.41737 \cdot 10^4$
Improper Dihedral	$2.22916 \cdot 10^2$	$2.22916 \cdot 10^2$
LJ-14	$7.31825 \cdot 10^3$	$7.31838 \cdot 10^3$
Coulomb-14	$7.09673 \cdot 10^3$	$7.09682 \cdot 10^3$
LJ (SR)	$5.15121 \cdot 10^4$	$5.15121 \cdot 10^4$
Coulomb (SR)	$-1.45845 \cdot 10^5$	$-1.45845 \cdot 10^5$
Coulomb Reciprocal	$6.23127 \cdot 10^3$	$6.23134 \cdot 10^3$
Position Restraints	$5.17674 \cdot 10^{-4}$	$5.17674 \cdot 10^{-4}$
Dihedral Restraints	$2.60336 \cdot 10^2$	$2.60336 \cdot 10^2$
Potential Energy	$4.37845 \cdot 10^3$	$4.37829 \cdot 10^3$
Pressure (bar)	$3.58884 \cdot 10^4$	$3.58904 \cdot 10^4$
Constraint RMSD	$2.08846 \cdot 10^{-6}$	$2.08842 \cdot 10^{-6}$

From visual inspection (Table S1), it is clear that the numbers are no longer identical. From this, it is assumed that back-conversion energies would rapidly differ if errors were made in the mapping.

Simulation set-up

1. **Phenylacetic acid:**

CHARMM .psf and .crd files for phenylacetic acid were obtained via CHARMM-GUI's Ligand reader. These were then converted to CHARMM36m and AMBER (GAFF2) via CHARMM-GUI's Force Field Converter.

2. **Human serum albumin:**

CHARMM-GUI's PDB reader was used to obtain CHARMM36m and AMBER ff19SB parameters. Default options were used. It should be noted that human serum albumin is a monomeric protein, however, the crystal structure contains 2 chains.

3. **Viral membrane**

The viral membrane was obtained from CHARMM-GUI's archive

"CHARMM-GUI Archive - COVID-19 Proteins Library" from the 6VSB 1_1_1 tarball. The membrane was extracted via gmx editconf and an index file.

4. **Fully glycosylated protein**:

The 6VSB 1_1_1 PDB was downloaded from the same archive as the viral membrane. This was then read through CHARMM-GUI's PDB reader using the default options.

Abbreviations used in Table 2

Table S2: Abbreviations for the molecules

**Abbreviation**	**Description**
BZAA	Phenylacetic acid
CHL	Cholesterol
DPPC	1,2-Dipalmitoyl-sn-glycero-3-phosphocholine
PSM	Palmitoyl sphingomyelin (N-palmitoyl-sphingomyelin)
DPPE	1,2-Dipalmitoyl-sn-glycero-3-phosphoethanolamine
POPE	1-Palmitoyl-2-oleoyl-sn-glycero-3-phosphoethanolamine
POPC	1-Palmitoyl-2-oleoyl-sn-glycero-3-phosphocholine
POPS	1-Palmitoyl-2-oleoyl-sn-glycero-3-phospho-L-serine
DPPS	1,2-Dipalmitoyl-sn-glycero-3-phospho-L-serine
POT	Potassium
CLA	Chloride
TIP3P	3-point water model (Transferable Intermolecular Potential with 3 Points)
PROA	Protein chain A
PROB	Protein chain B

Test case: (Lack of) effect of incorrect hydrogen ordering

The phenylacetic acid previously generated contains 2 hydrogens on carbon 12 (name C7), making it a good test case to see how hydrogen ordering affects the system.

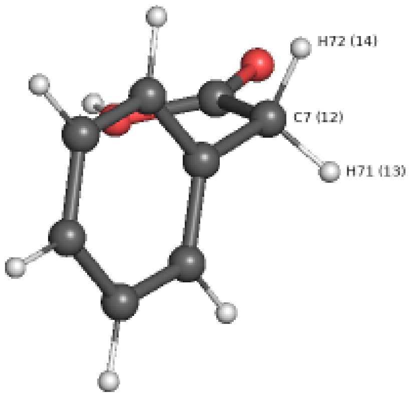

Figure S3: Image of phenylacetic acid.

From a visual inspection (Fig. S3), it is clear that the hydrogens are chemically equivalent. By examining the topology (obtained by grep “13” and grep “14”) the hydrogens are present in the following lines.

```
13 HGA2 1 BZAA H71 13 0.090000 1.0080 ; qtot -0.090
12 13 1
 7 13 1
10 13 1
13 16 1
13 17 1
 9 12 13 5
13 12 14 5
13 12 15 5
 7 9 12 13 9
10 9 12 13 9
13 12 15 16 9
13 12 15 17 9
```

```
14 HGA2 1 BZAA H72 14 0.090000 1.0080 ; qtot 0.000
14 12 1
 7 14 1
10 14 1
14 16 1
14 17 1
 9 12 14 5
13 12 14 5
14 12 15 5
 7 9 12 14 9
10 9 12 14 9
14 12 15 16 9
14 12 15 17 9
```

From the atoms block (first line), it is confirmed that the hydrogens are chemically equivalent as they share the same atom type. Comparing the other lines, each line is present once, with the only substitution being 13 being substituted to 14 and vice-versa. In this case, Gromacs will essentially replace the first hydrogen by another hydrogen that follows the same criteria, the incorrect ordering does not impact anything. The single point energy of phenylacetic acid with the hydrogens shuffled remains the same.

3. User manual for gmx_ffconv

Installation instructions

As gmx_ffconv is available through PyPi, the recommended installation instructions are to simply install with pip:

```
pip install gmx_ffconv
```

pypi v1.0.2

Alternatively, the GitHub contains the latest development version:

`https://github.com/Jassu1998/gmx_ffconv`

Example usage of gmx_ffconv

Tips for use

(1) If you are working on very large molecules, the default memory stack allocation can lead to a segfault error. This can easily be fixed by running

```
ulimit -s unlimited
```

in the same terminal before invoking gmx_ffconv.

(2) When building a database of mappings, for example from AMBER's Lipid21 to CHARMM36m, storing the folder with the included topology files for the new force field inside the mappings directory enables groconv to find them without having to manually provide paths.

Examples

The files are included inside the "Tutorial" folder in the zip file included with the manuscript.

Example 1: Small membrane

A membrane containing 24 DOPE, 24 DPPC, 24 CHL, 6 K+, 6 Cl- and 2709 TIP3P molecules was generated by CHARMM-GUI's Membrane builder using CHARMM36m. The target force field is AMBER (Lipid21). The water model is kept as TIP3P, and the ions are standard for the water model.

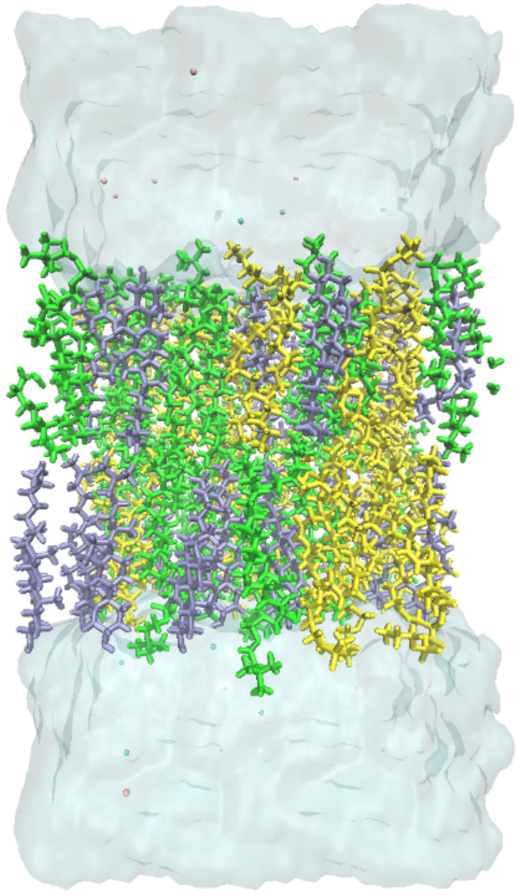

Figure S4: Membrane (CHARMM36m) containing DPPC (green), cholesterol (blue), DOPE (yellow).

First, mappings for each molecule must be found. These commands can be run at the same time by the use of & at the end of each command.

DPPC

```
gmx_ffconv ffmap -itp1 toppar_CHARMM/DPPC.itp -itp2 toppar_AMBER/PPPC.itp -name DPPC
```

Cholesterol

```
gmx_ffconv ffmap -itp1 toppar_CHARMM/CHL1.itp -itp2 toppar_AMBER/CHL.itp -name CHL
```

DOPE

```
gmx_ffconv ffmap -itp1 toppar_CHARMM/DOPE.itp -itp2 toppar_AMBER/OOPE.itp -name DOPE
```

TIP3P

```
gmx_ffconv ffmap -itp1 toppar_CHARMM/TIP3.itp -itp2 toppar_AMBER/TP3.itp -name TIP3P
```

Potassium

```
gmx_ffconv ffmap -itp1 toppar_CHARMM/POT.itp -itp2 toppar_AMBER/K+.itp -name POT
```

Chloride

```
gmx_ffconv ffmap -itp1 toppar_CHARMM/CLA.itp -itp2 toppar_AMBER/Cl-.itp -name CLA
```

These should have generated multiple mapping files, one mapping file per molecule type named mapping_NAME.csv.Now, that all mappings are obtained, the new coordinate file can be obtained via groconv. **Note: The order of names provided must match the ordering used in the coordinate file.**

```
gmx_ffconv groconv -coordfile CHARMM_MEMB.gro \
-name DOPE DPPC CHL POT CLA TIP3P \
-nmol 24 24 24 6 6 2709 \
-output AMBER_MEMB_from_CHARMM.gro
```

We can now compare the AMBER_MEMB_from_CHARMM.gro to AMBER_MEMB_FF.gro, which will match in energy values if the conversion has been successful. This file is provided in the AMBER_REF folder with the necessary files.

Example 2: Phenylacetic acid + Membrane

Now, we find ourselves wanting to use the previously generated membrane with a phenylacetic acid added to the system.

However, we have already mapped out the lipids (DPPC, DOPE, and CHL), the solvent (TIP3P) and the counterions (potassium and chloride). Instead of carrying out ffmap again, it is recommend to copy the mappings in a mapping database, and include the folder with the included topology files and copy them in the folder of example 2.

```
mkdir MAPPINGS_CHARMM_TO_AMBER
cp mapping*.csv MAPPINGS_CHARMM_TO_AMBER
cp -r toppar_AMBER MAPPINGS_CHARMM_TO_AMBER
cp -r MAPPINGS_CHARMM_TO_AMBER ../Example2/
```

We should now navigate into the Example2 folder and obtain the mapping for phenylacetic acid from CHARMM to AMBER.

```
gmx_ffconv ffmap -itp1 toppar_CHARMM/BZAA.itp -itp2 toppar_AMBER/BZAA.itp -name BZAA
```

We can move the new mapping for BZAA to the database.

```
cp mapping_BZAA.csv MAPPINGS_CHARMM_TO_AMBER/
```

The new system with phenylacetic acid has the following number of molecules in the topology :

```
BZAA  1
DOPE  24
DPPC  24
CHL1  24
POT  30
CLA  30
TIP3  8925
```

From this, we obtain the following command for groconv:

```
gmx_ffconv groconv -coordfile BZAA_MEMB_CHARMM.gro \
  -nmol 1 24 24 24 30 30 8925 \
  -name BZAA DOPE DPPC CHL POT CLA TIP3P \
  -output BZAA_MEMB_AMBER_from_CHARMM.gro \
  -mapping_dir MAPPINGS_CHARMM_TO_AMBER
```

We can now compare the converted to the reference AMBER configuration obtained from CHARMM-GUI.

Table S3: Comparison of energy components between converted structure using gmx_ffconv and CHARMM-GUI's Force Field Converter.

**Energy Component**	**Amber Converted**	**Amber Reference**
Bond	$9.40996 \cdot 10^{3}$	$9.40996 \cdot 10^{3}$
Urey-Bradley	$5.13779 \cdot 10^{4}$	$5.13779 \cdot 10^{4}$
Proper Dihedral	$1.52890 \cdot 10^{4}$	$1.52890 \cdot 10^{4}$
Periodic Improper Dihedral	$3.62263 \cdot 10^{2}$	$3.62263 \cdot 10^{2}$
LJ-14	$1.13004 \cdot 10^{4}$	$1.13004 \cdot 10^{4}$
Coulomb-14	$-5.06036 \cdot 10^{3}$	$-5.06036 \cdot 10^{3}$
LJ (SR)	$6.84015 \cdot 10^{20}$	$6.84015 \cdot 10^{20}$
Dispersion Correction	$-7.14058 \cdot 10^{3}$	$-7.14058 \cdot 10^{3}$
Coulomb (SR)	$2.50072 \cdot 10^{6}$	$2.50072 \cdot 10^{6}$
Coulomb Reciprocal	$3.12424 \cdot 10^{4}$	$3.12424 \cdot 10^{4}$
Position Restraints	$8.64137 \cdot 10^{-3}$	$8.64137 \cdot 10^{-3}$
Dihedral Restraints	$2.06953 \cdot 10^{2}$	$2.06953 \cdot 10^{2}$
Potential	$6.84015 \cdot 10^{20}$	$6.84015 \cdot 10^{20}$
Pressure DC (bar)	$-1.39720 \cdot 10^{3}$	$-1.39720 \cdot 10^{3}$
Pressure (bar)	$2.93140 \cdot 10^{21}$	$2.93140 \cdot 10^{21}$
Constraint RMSD	$1.88049 \cdot 10^{-6}$	$1.88056 \cdot 10^{-6}$

All of the energies match (Table S3), except constraint rmsd, which comes from the LINCS constraint solver, this difference can be ignored), indicating successful conversion.

Example 3: Converting DPPC from AMBER to CHARMM with consistent naming

In this example, the aim is to convert an AMBER Lipid21-formatted DPPC coordinate file to CHARMM36m . However, unlike previously, this time consistent atom naming across force fields is maintained for the tail hydrogens. DPPC (AMBER Lipid21, DPPC) uses the same atom naming convention for the hydrogens in both tails (R,S), whereas DPPC (CHARMM36m) uses different ones (R,S and X,Y in each tail). If the interest is to obtain a mapping for the first tail, where the atom names are the same, for example H2S and H2S, then the following CSV (DPPC_tail1.csv) will generate the desired mapping.

```
AMBER,CHARMM
H2S,H2S
H2R,H2R
H3R,H3R
H3S,H3S
H4S,H4S
H4R,H4R
H5S,H5S
H5R,H5R
H6S,H6S
H6R,H6R
H7S,H7S
H7R,H7R
H8S,H8S
H8R,H8R
H9R,H9R
H9S,H9S
H10S,H10S
H10R,H10R
H11S,H11S
H11R,H11R
H12S,H12S
H12R,H12R
H13S,H13S
H13R,H13R
H14S,H14S
H14R,H14R
H15S,H15S
H15R,H15R
H16T,H16T
H16R,H16R
H16S,H16S
```

However, the other tail might be off interest as well. In this case, atom names must be made unambiguous, which is done here by the addition of an underscore to the atom names

in AMBER. The underscores must also be added to the AMBER itp file (PPPC.itp). In this case they must be added to the first PA residue in the AMBER topology file. As the new force field file (CHARMM_DPPC.itp) was not altered, no further steps are required and we can proceed as standard with groconv. If the itp that required modification was the new itp file, then for groconv users should provide the original, unaltered one by changing the path in the mapping CSV file.

Snippet of AMBER itp [atoms] section with renamed atoms (AMBER_DPPC.itp):

```
[ atoms ]
; nr type resnr residu atom cgnr charge mass
    1 cD 2 PA C116 1 -0.125447 12.0100 ; qtot -0.125
    2 hL 2 PA H16R_ 2 0.029047 1.0080 ; qtot -0.096
    3 hL 2 PA H16S_ 3 0.029047 1.0080 ; qtot -0.067
    4 hL 2 PA H16T_ 4 0.029047 1.0080 ; qtot -0.038
    5 cD 2 PA C115 5 0.013975 12.0100 ; qtot -0.024
    6 hL 2 PA H15R_ 6 0.009292 1.0080 ; qtot -0.015
    7 hL 2 PA H15S_ 7 0.009292 1.0080 ; qtot -0.006
    8 cD 2 PA C114 8 -0.020086 12.0100 ; qtot -0.026
    9 hL 2 PA H14R_ 9 0.015929 1.0080 ; qtot -0.010
   10 hL 2 PA H14S_ 10 0.015929 1.0080 ; qtot 0.006
   11 cD 2 PA C113 11 -0.033096 12.0100 ; qtot -0.027
   12 hL 2 PA H13R_ 12 0.014621 1.0080 ; qtot -0.012
   13 hL 2 PA H13S_ 13 0.014621 1.0080 ; qtot 0.002
   14 cD 2 PA C112 14 -0.027633 12.0100 ; qtot -0.025
   15 hL 2 PA H12R_ 15 0.011368 1.0080 ; qtot -0.014
   16 hL 2 PA H12S_ 16 0.011368 1.0080 ; qtot -0.003
   17 cD 2 PA C111 17 -0.025206 12.0100 ; qtot -0.028
   18 hL 2 PA H11R_ 18 0.014334 1.0080 ; qtot -0.014
   19 hL 2 PA H11S_ 19 0.014334 1.0080 ; qtot 0.001
   20 cD 2 PA C110 20 -0.028831 12.0100 ; qtot -0.028
   21 hL 2 PA H10R_ 21 0.014691 1.0080 ; qtot -0.013
   22 hL 2 PA H10S_ 22 0.014691 1.0080 ; qtot 0.001
   23 cD 2 PA C19 23 -0.030472 12.0100 ; qtot -0.029
   24 hL 2 PA H9R_ 24 0.013897 1.0080 ; qtot -0.015
   25 hL 2 PA H9S_ 25 0.013897 1.0080 ; qtot -0.001
   26 cD 2 PA C18 26 -0.015793 12.0100 ; qtot -0.017
   27 hL 2 PA H8R_ 27 0.009067 1.0080 ; qtot -0.008
   28 hL 2 PA H8S_ 28 0.009067 1.0080 ; qtot 0.001
   29 cD 2 PA C17 29 -0.019630 12.0100 ; qtot -0.019
   30 hL 2 PA H7R_ 30 0.011041 1.0080 ; qtot -0.008
```

If one wanted to simply add an underscore to all atom names present in the first PA residue in the AMBER topology, this is also supported.

By visually inspecting the CHARMM and AMBER DPPC coordinate files (DPPC_CHARMM.gro and DPPC_AMBER.gro), we find that S,R,T in AMBER are equivalent to Y,X and Z in CHARMM. We get the following equivalencies for hydrogens present in the second tail.

```
H2S_,H2Y
H3R_,H3X
H3S_,H3Y
H4S_,H4Y
H4R_,H4X
H5S_,H5Y
H5R_,H5X
H6S_,H6Y
H6R_,H6X
H7S_,H7Y
H7R_,H7X
H8S_,H8Y
H8R_,H8X
H9S_,H9Y
H9R_,H9X
H10S_,H10Y
H10R_,H10X
H11S_,H11Y
H11R_,H11X
H12S_,H12Y
H12R_,H12X
H13S_,H13Y
H13R_,H13X
H14S_,H14Y
H14R_,H14X
H15S_,H15Y
H15R_,H15X
H16S_,H16Y
H16R_,H16X
H16T_,H16Z
```

By adding the above snippet (DPPC_tail2_noheader.csv) to the previous CSV , both tails will follow atom naming consistent ordering.

```
awk 1 DPPC_tail1.csv DPPC_tail2_noheader.csv > DPPC_tail1_tail2.csv
```

Now that the CSV file is ready and the AMBER topology has been altered, we can run

```
gmx_ffconv ffmap -itp1 AMBER_DPPC.itp -itp2 CHARMM_DPPC.itp -name DPPC \
--consistent_naming DPPC_tail1_tail2.csv
```

The timing of ffmap with these additional hydrogen constraints has noticeably improved, and is only taking approximately 0.1 seconds instead of around a minute when only chemical elements and connectivity are considered.

We can now simply convert as standard using groconv:

```
gmx_ffconv groconv -coordfile DPPC_AMBER.gro -name DPPC \
-output DPPC_CHARMM_converted.gro -nmol 1
```
